# Shaping Tomorrow: The Vision Ahead for *IJPS*

**DOI:** 10.1055/s-0045-1804534

**Published:** 2025-03-11

**Authors:** Maneesh Singhal

**Affiliations:** 1 Department of Plastic, Reconsrtuctive and Burns Surgery, All India Institute of Medical Sciences, New Delhi, India

As we usher in 2025, I extend my sincere wishes to all members of the *Indian Journal of Plastic Surgery (IJPS)* community. It is with immense pride that I assume the role of Editor-in-Chief for the Indian Journal of Plastic Surgery (IJPS). I am filled with gratitude and a profound sense of responsibility as we embark on this new chapter. This year not only marks a fresh beginning for me but also represents a pivotal moment for our journal as we undertake an ambitious journey over the next three years to enhance our contributions to the field of plastic surgery.

Established in 1968, IJPS has been a cornerstone of plastic surgery literature for over five decades. Our recent achievement, attaining our first impact factor, is a testament to the dedication of our contributors and reviewers. IJPS is distinguished as one of the first three journals globally dedicated to the specialty of plastic surgery. 1 2 As the first Asian journal in this field, it has positioned India at the forefront of scientific publishing, surpassing many regions, including Europe and Australia. From its inception, the Indian Journal of Plastic Surgery (IJPS) has upheld its mission of disseminating high-quality scientific knowledge without financial barriers. The journal is indexed in PubMed and operates under a diamond open-access model, ensuring that authors incur no costs for publishing their work while readers enjoy unrestricted access. 3 This commitment to accessibility reflects our dedication to promote innovation and collaboration across the global plastic surgery community.

The *IJPS* serves as the official journal of the Association of Plastic Surgeons of India (APSI). The journal has evolved alongside the APSI, reflecting the society's progress. With an impact factor of 0.7, the journal enjoys a respected position globally; yet there remains significant, untapped potential to further enhance its stature.

As we move forward, our commitment to enhancing the quality and visibility of IJPS will be paramount.The remarkable journey of IJPS is built on the vision and dedication of its previous editors. Their tireless efforts have brought the journal to its current standing as a trusted and respected platform. I extend my heartfelt appreciation to my predecessors, whose hard work and vision have laid a solid foundation for IJPS. I extend my deep gratitude to these pioneers, who have left a legacy of excellence and innovation. Their commitment to advancing knowledge in plastic surgery has been truly inspiring. I am especially thankful to the Association of Plastic Surgeons of India (APSI) for their unwavering support, which has propelled our journal to new heights.

The journal has grown alongside APSI, serving as a reflection of our society's progress. It is now a respected source of knowledge and collaboration. As I assume this role, I am inspired to build on this strong foundation and lead IJPS into an era of even greater impact and influence.

My vision for IJPS is rooted in three guiding principles: enhancing quality, broadening reach, and fostering inclusivity.

To achieve these goals, I plan to implement the following initiatives:

- **Enhanced online presence:** We aim to leverage social media and other digital platforms to promote published articles and special issues. To support this, we will include dedicated social media team, tasked with creating engaging content and maximizing audience outreach.
- **Optimizing editorial processes:** We are streamlining the peer review process to make it faster and more efficient. Desk rejections or reviewer assignments will be completed within 3 weeks, and the entire review process will be completed within 4 weeks. Once accepted, articles will be published within 3 weeks, and we will aim for PubMed indexing within a month.
  To strengthen our peer review process, we encourage active participation from the global plastic surgery community. Reviewers will be recognized on platforms like ORCID and Publons, and top contributors will be awarded annually. Exceptional reviewers will also have the opportunity to join our editorial board where they can help drive the journal's strategic vision.
- **Building a strong editorial team:** We shall aim to develop a diverse and dynamic editorial board ensuring alignment between members' research expertise and the journal's objectives.
- **Special issue campaigns:** We intend to introduce “Spotlight on Emerging Trends in Plastic Surgery”—an innovative set of special issues that will showcase the most recent, groundbreaking topics on the subject. Proposals for special issues shall be invited from our editorial board and the broader research community. Approved proposals will receive comprehensive support, including assistance in concept development and crafting compelling Calls for Papers to attract high-quality submissions.
- **Supporting authors:** A key priority is to encourage a diverse range of submissions from both established and emerging researchers. The Indian plastic surgery community is brimming with unique clinical insights that deserve broader recognition. My goal would be to foster an environment where authors feel supported in sharing their work, particularly in areas where India has notable expertise but has limited representation in published literature.
- **Upholding ethical standards:** The *IJPS* is committed to strictly adhering to the guidelines established by the Committee on Publication Ethics (COPE) and ensuring that all authors understand their responsibilities regarding ethical research practices. This includes transparency in reporting conflicts of interest and safeguarding patient confidentiality in all published studies. Having IEC approval for all original articles will be mandatory before proceeding with peer review.
- **Go green initiative:** Committed to global sustainability, we are launching a Go Green initiative to minimize our environmental footprint. By championing sustainability, we aim to make a positive impact on both our field and the planet.

In addition to these initiatives, I am committed to expanding the journal's global presence, and fostering international collaboration.

The success of such vision depends on the collective efforts of authors, reviewers, readers, and the editorial team. Together, we can sustain and grow the *IJPS* as a platform for innovation, learning, and collaboration in the field of plastic surgery globally.

I sincerely thank you all for your trust and support and look forward to working together to elevate the *IJPS* to new heights.
